# Utility of 18-Flurodeoxyglucose Positron Emission Tomography-Computed Tomography ( ^18^ FDG PET-CT) in Gallbladder Cancer: Experience from a Tertiary Care Hospital

**DOI:** 10.1055/s-0043-1777699

**Published:** 2023-12-26

**Authors:** Niharika Bisht, Nishant Lohia, Sankalp Singh, Arti Sarin, Abhishek Mahato, Dharmesh Paliwal, Indranil Sinha, Sharad Bhatnagar

**Affiliations:** 1 Radiation Oncology, Army Hospital (RR), Delhi, India; 2 Radiation Oncology, Assam Cancer Care Foundation (ACCF), Kokrajhar, Assam, India; 3 Nuclear Medicine Specialist, Command Hospital (CC), Lucknow, Uttar Pradesh, India; 4 Radiation Oncology, ESI Medical College, Faridabad, Haryana, India

**Keywords:** gallbladder cancer, PET-CT, staging, response assessment, metastatic

## Abstract

**Introduction** Gallbladder cancer (GBC) is one of the most common and aggressive malignancies of the Indo-Gangetic plains. Despite its widespread use in GBC cases, the role of 18-flurodeoxyglucose positron emission tomography-computed tomography ( ^18^ FDG PET-CT) in the management of this disease is not well defined. In our study, we present the practice trends of the utilization of this investigative modality in our hospital and its benefits in aiding diagnosis, staging, and surveillance for recurrence.

**Materials and Methods** All cases of suspected and biopsy-proven GBCs who underwent PET-CT at our institute between 2016 and 2019 were retrospectively evaluated for the indication of PET-CT testing and its impact on the management of the case. The indications were classified into three categories: (i) staging and metastatic workup, (ii) response assessment post-chemotherapy, and (iii) post-therapy surveillance of patients.

**Results** A total of 79 PET-CT scans were carried out during the study period. PET-CT was used for less than one-third of the total patients of GBC presenting at our center. Initial staging and workup (49%) was the most common indication followed by surveillance (28%) and response assessment (23%). PET-CT had a substantially better sensitivity in detecting distant metastases compared to conventional imaging in both initial workup and during follow-up. PET-CT provided additional information in 42% scans that led to change in the management of the patient. As a response assessment tool PET-CT aided not only in evaluating efficacy of therapy but also for documenting progressive disease for patients on therapy.

**Conclusion** PET-CT is a valuable tool to not only rule out metastatic disease while selecting patients for surgery but also for post-therapy surveillance for recurrence in patients of GBC. Larger prospective studies may help in finally elucidating the exact role of PET-CT in this disease.

## Introduction

Gallbladder cancer (GBC) is a relatively rare malignancy having a worldwide prevalence of less than 2 per 100,000, though it is the commonest cancer of the biliary tract. In India, its demographic picture is characterized by marked geographical and ethnic variations, with very high incidence rates in North-Eastern and Central India and contrastingly low incidence rates in Southern and Western India. 1 It is an aggressive malignancy with 5-year survival rates of less than 5% and an overall mean survival rate as low as 6 months, 2 primarily because of an insidious onset and gradual progression through metaplasia-dysplasia-adenocarcinoma. This results in late diagnoses in locally advanced or metastatic stages of the majority of the cases. 2 3 The most common histological type is adenocarcinoma not-otherwise-specified (NOS). However, the most favorable type is the papillary type due to its propensity to spread intraluminally, unlike other types that spread transmurally and become invasive. Carcinoma of the gallbladder is associated with de novo genetic changes due to p53 alterations associated with a low percentage of K-ras mutations. It also shows an adenoma-carcinoma sequence in the absence of p53, K-ras or APC gene mutations. 4 GBC is an aggressive disease with common local invasion, early and widespread nodal metastases, and frequent distant metastases. Presenting symptoms are often misdiagnosed as biliary colic or chronic cholecystitis, further delaying diagnosis. 5 6

According to the 8th edition of the American Joint Committee on Cancer (AJCC) classification, the T2 classification now differentiates between the peritoneal and hepatic surfaces (T2a and T2b, respectively). T3 tumors perforate the GB serosa or penetrate into the liver or one other adjacent organ. T4 tumors are defined as those that invade the main portal vein, hepatic artery, or tumors that invade 2 or more extrahepatic organs. The updated N-category is defined by the number of metastatic lymph nodes (LN; N1 5 1–3 LN metastases, N2 5 4 or more LN metastases), instead of their anatomic position. In a node-negative setting, T1 tumors are stage I; T2a tumors are stage IIA; T2b tumors are stage IIB; and T3 tumors are stage IIIA. T3N1 disease is defined as stage IIIB. Stage IV tumors include all T4 lesions (stage IVA), all N2 diseases (stage IVB), and all metastatic diseases (stage IVB). 7 8

Curative surgery with R0 resection is the mainstay of treatment in this malignancy. However, only 15 to 20% of these patients are surgical candidates due to local infiltration by the disease or distant metastasis. Among locally advanced cases, significant morbidity is suffered by nearly 50% of patients undergoing extensive surgery and 5-year survival even after radical surgery is less than 20%. 8 9 10 Hence, it is vital to select only those candidates for surgery who may potentially benefit from it. Radiotherapy and chemotherapy are being explored in a neoadjuvant manner to downstage the disease and to select favorable, nonprogressive diseases for surgical resection. 11 12 The accurate assessment of response to these therapies is also crucial in deciding further management of the patient.

With this background, comprehensive and accurate staging becomes an imperative part of treatment, especially to rule out distant metastasis before deciding on the intent of therapy. Multiphasic abdominopelvic CT/MRI (magnetic resonance imaging) followed by staging laparoscopy is the current standard of care. 13 14 Though there are studies supporting the role of positron emission tomography computed tomography (PET-CT) in GBC, in cases incidentally detected at the time of post-surgical histopathology and in those diagnosed radiologically before surgery, 15 16 17 18 there is a lack of consensual support in defining the role of PET-CT in GBC, especially in locally advanced cases. There is also limited literature support exploring the role of PET-CT as a tool for response assessment post-therapy. Nevertheless, PET-CT is used widely by clinicians, extrapolating guidelines of other malignancies like gastrointestinal cancers and breast cancer where it is an established staging tool. We present a single-center experience exploring the utility of PET-CT in the staging, management, and post-therapy follow-up of GBCs.

## Materials and Methods

This was a retrospective study where we evaluated medical records of 79 cases of GBCs who underwent PET-CT at our institute between 2017 and 2019. This study was carried out at an oncology department of a tertiary cancer care center of Northern India and was approved by the institutional review board. It included biopsy-proven or suspected GBC patients (suspected refers to patients who did not have histological proof of malignancy at the time of PET-CT but were confirmed subsequently). Included patients were either treatment-naive and under initial evaluation, or mid-treatment, or post-therapy (surgery, chemotherapy, or radiotherapy). After evaluation of the treatment records and the PET-CT reports, the cases were classified into the following three groups depending upon the indication of the PET-CT: (i) initial staging and metastatic workup, (ii) response assessment post-chemotherapy, and (iii) surveillance or follow-up.

All patients underwent PET-CT in the nuclear medicine department of our institute where the nuclear medicine expert interpreted the imaging and discussed the findings with the multidisciplinary oncology team. The findings were interpreted based on 18-flurodeoxyglucose (FDG) avidity and differential contrast enhancement. Values of maximum standardized uptake value (SUV)max more than 2.4 units were usually considered significant and positive based on the study by Goel et al. 16 However, any lesion with a SUVmax below this value but with presence of morphological features suspicious of malignancy in the opinion of nuclear medicine physician/radiologist was also considered significant and recommended for cytological or histological sampling.

The PET-CT study was deemed negative if there were no suspicious lesions or any abnormal ^18^ FDG SUV values crossing the mentioned value. All patients were staged as per the AJCC 8th edition. 19 For simplicity of comparison, patients were staged into local (T1-T4, N0M0), locoregional (any T, N1-N2, M0), and metastatic (any T, any N, M1). Any suspicion of local recurrence or distant metastases on conventional or functional imaging was histopathologically confirmed by sampling of tissue from suspected site. Histopathological confirmation of malignancy was treated as the gold standard of testing. The PERCIST criteria were used to grade the treatment response on PET-CT. 20 Data about the use of tumor markers in our study subjects was not uniformly or completely available and hence was not presented or analyzed.

After collection and categorization of PET-CT results and treatment data, it was analyzed to see if the use of PET-CT in each case had (i) added any new information to the case in terms of staging or response to treatment and (ii) if the added information had led to any change in the final management of that particular cases compared to the conventional imaging investigations.

## Results

A total of 79 scans of GBC patients were undertaken at our institute during the study period. Out of these, the majority of patients (59; 75%) were females, while 20 (25%) patients were males. The age of the patients ranged from 22 to 80 years with a median age of 56 years. The demographic data along with stage distribution and treatment history is depicted in Table 1 .

**Table 1:** Demographic and clinical data of study sample

Parameter	Frequency (% or range)
Age	
Range	22–80 years
Median	56 years
Sex	
Female	59 (75)
Male	20 (25)
Stage	
Local	15 (19)
Locoregional	31 (39)
Metastatic	33 (42)
Treatment history	
Chemotherapy (as neoadjuvant, adjuvant, or palliative)	64 (81%)
No chemotherapy	15 (19%)
Surgery	37 (47%)
Radical cholecystectomy	25 (32%)
Simple cholecystectomy	12 (15%)
No surgery	42 (53%)
Radiotherapy	2 (2.5%)
No radiotherapy	77 (97.5%)

The range of SUVmax for primary lesions was from 3.42 to 23.79 with a mean of 14.8. In metastatic lesions, the range of SUVmax was from 3.8 to 13.1 with a mean of 8. In all except two cases, the SUVmax of the primary was higher than that of the metastatic sites. The radioisotope uptake pattern of the primary lesion allowed invasion of local structures (liver, extra-hepatic bile ducts, vessels, duodenum, and colon) to be discernable in 24 (30.38%) cases, while regional lymph nodal involvement was seen in 29 (36.71%) cases.

The different histological subtypes encountered in the study were adenocarcinomas NOS—64 (81%), papillary adenocarcinoma—5 (6.3%), mucinous adenocarcinoma—3 (3.8%), adenosquamous carcinoma—3 (3.8%), undifferentiated type—2 (2.5%), and small cell carcinoma—1 (1.3%). Due to the significant variation in the number of cases of adenocarcinoma NOS and other subtypes, no statistical testing of average SUVmax values could be carried out. However, the highest SUVmax was seen in an adenosquamous carcinoma (23.79), while the lowest was seen in a papillary carcinoma (3.42).

**Fig. 1:**
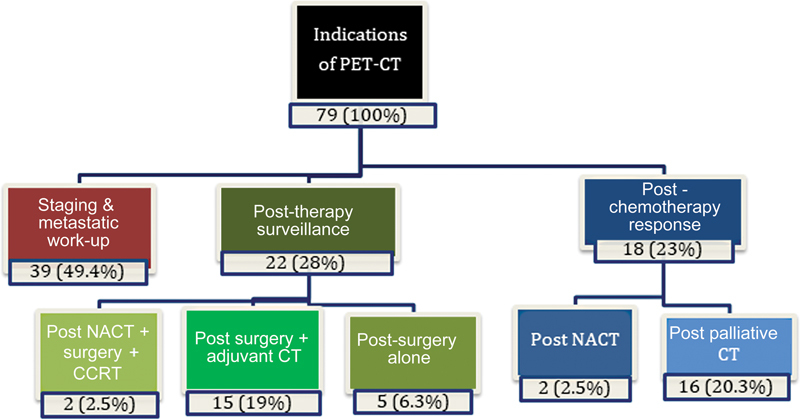
Distribution of indications of positron emission tomography-computed tomography (PET-CT) in carcinoma gallbladder patients in our study. NACT, neoadjuvant chemotherapy.

**Fig. 2:**
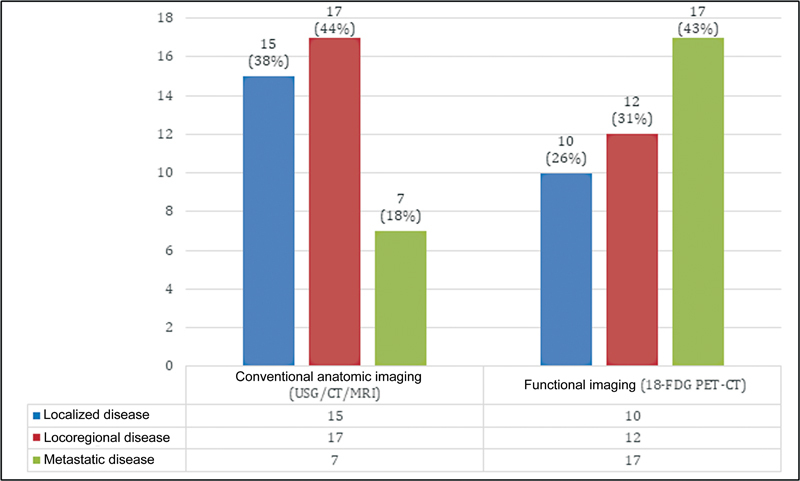
Difference in disease staging between conventional and functional imaging in 39 cases where positron emission tomography-computed tomography (PET-CT) was used for initial staging of disease. Ten (26%) additional patients were found to be metastatic on PET-CT compared to conventional imaging. USG, ultrasonography.

The varied indications for which PET-CT was done are summarized in Fig. 1 . The most common indication for carrying out PET-CT in our study was for initial staging and metastatic workup that was done in 39 cases (49%). The change in stage of disease post-functional imaging in the form of PET-CT as compared with anatomical imaging in the form of ultrasonography (USG), CT, or MRI is depicted in Fig. 2 . As seen in the figure, PET-CT was better than conventional imaging during staging for the detection of distant metastases. Ten (26%) additional cases were found to be metastatic on PET than conventional imaging. Two more cases detected to be metastatic on PET-CT did not show any signs of malignancy on histopathological examination of metastatic sites. On statistical analysis, PET had a sensitivity of 100% and a specificity of 90.9% for detecting distant metastases while staging patients per primum. Conventional imaging, on the other hand, had a sensitivity of only 41.12% and a specificity of 100% for the detection of distant metastases during staging workup. As all patients did not undergo surgery, the confirmation of local and locoregional staging by PET-CT by histopathological examination of the specimen could not be done. Therefore, statistical comparison of functional and metabolic imaging could not be done for local or locoregional staging.

**Fig. 3:**
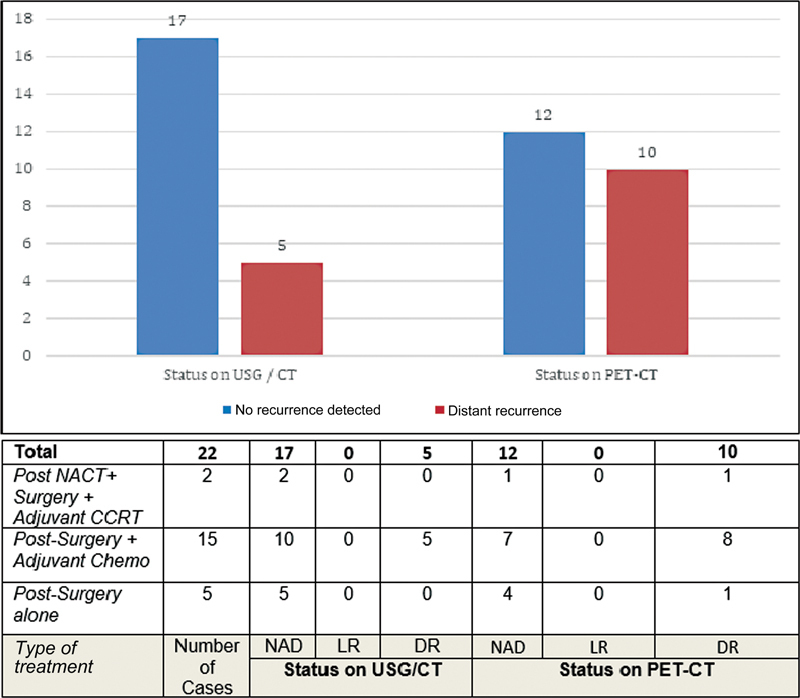
Difference in disease status during surveillance by conventional (USG/CT) versus functional (PET-CT) imaging in the 22 cases, where PET-CT was used for surveillance for distant recurrence. Five (22.7%) additional patients were found to be metastatic on PET-CT. CCRT, concurrent chemoradiotherapy; DR, distant recurrence; LR, local recurrence; NACT, neoadjuvant chemotherapy; NAD, no abnormality detected; PET-CT, positron emission tomography-computed tomography; USG, ultrasonography.

PET-CT scan, done for post-treatment surveillance and follow-up, was the second-commonest indication in our study and was carried out in 22 cases (28%). Fig. 3 displays the difference in disease status on surveillance using conventional imaging (USG or CT) versus functional imaging (PET-CT). PET-CT upstaged the disease by detecting distant metastases in five cases (22.7%) that had not been picked up on conventional imaging. On statistical analysis, PET-CT had a sensitivity of 100% for the detection of distant metastases during follow-up compared to only 50% for conventional imaging. The specificity for both types of imaging for detection of distant metastases was 100%. Interestingly, no local recurrences occurred or were picked up by either PET-CT or conventional imaging in this subset of 22 patients.

**Fig. 4:**
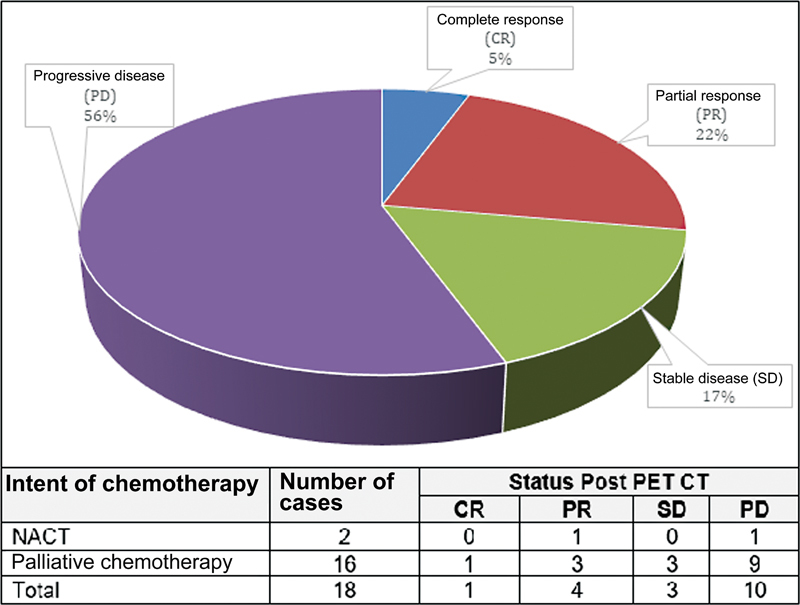
Results of post-chemotherapy response assessment using 18-flurodeoxyglucose positron emission tomography-computed tomography ( ^18^ FDG PET-CT). CR, complete response; NACT, NACT, neoadjuvant chemotherapy; PD, progressive disease; PR, partial response; SD, stable disease.

Eighteen cases (23%) underwent PET-CT for response assessment post-chemotherapy. PET-CT was preferably and exclusively used in the assessment of treatment response in our hospital during the study period. No other modality of imaging or tumor markers was used in a “before” and “after” setting in this subset. Hence, there was no investigative modality to compare PET-CT in this indication. Results of PET-CTs done after therapy were compared with pre-chemotherapy PET-CTs done for disease staging. PET-CT was done in two cases post-neoadjuvant chemotherapy (NACT), where 1 case had partial response and the other had progression of disease on PET-CT. In 16 cases, PET-CT was done post-palliative chemotherapy and the disease status post-PET-CT in these 16 cases is reflected in Fig. 4 .

The use of PET-CT in GBC in our study gave additional information in almost 33 cases (42%) and thereby resulted in change of management in all these 33 cases. The impact of PET-CT resulting in newer findings and associated change in management in the varied indications as discussed above is summarized in Table 2 .

**Table 2:** Impact of use of PET-CT in GBC patients on their management in our study

	Total PET-CTs done	Additional information gained (%)	Change in management seen (%)
For staging	39	10 (26%)	10 (26%)
For surveillance	22	5 (23%)	5 (23%)
For response assessment	18	18 (100%)	18 (100%) a
Total	79	33 (42%)	33 (42%)

Abbreviations: GBC, gallbladder cancer; PET-CT, positron emission tomography-computed tomography.

a PET-CT was exclusively used for response assessment post-chemotherapy in our study subjects.

In our study, 17 cases were detected to have distant metastases on PET-CT. The distribution of metastases in all these 17 cases is shown in Table 3 .

**Table 3:** Distribution of metastatic sites detected on whole-body PET-CT

Metastatic sites on PET-CT	Liver	3
	Retroperitoneal lymph nodes	3
	Peritoneal deposits	2
	Retroperitoneal lymph nodes + liver	2
	Retroperitoneal lymph nodes + peritoneal deposits	2
	Retroperitoneal lymph nodes + peritoneal deposits + liver	2
	Retroperitoneal lymph nodes + mediastinal lymph nodes + lung	1
	Retroperitoneal lymph nodes + mediastinal lymph nodes + lung +supraclavicular lymph nodes + skeletal metastases	1
	Peritoneal deposits +Sister Mary Joseph (umbilical) nodule	1
	**Total**	**17**

Abbreviation: PET-CT, positron emission tomography-computed tomography.

## Discussion

Whole body ^18^ FDG PET-CT is routinely utilized as an investigative modality for staging and metastatic workup in most gastrointestinal malignancies, including esophageal, gastric, and colorectal cancers. 21 Despite its widespread use in staging, response assessment, and surveillance in cases of GBC, it is still not regarded as a standard of care investigation in this particular disease. 22 23 Many studies have employed the utility of PET-CT as a metastatic tool to identify lymph nodal and distant metastases and upstage the patients prior to radical surgery while comparing it in this role to contrast-enhanced computed tomography (CECT) abdomen. 15 16 24 However, there are no consensus guidelines regarding the role of PET-CT in the management of GBC. This is partly due to the high financial implications of the investigation and also the low incidence of this malignancy in Western counties where many such guidelines are generated.

Our results showed that in our institute, ^18^ FDG PET-CT was effectively used in the management of a sizable number of GBC cases in a variety of roles. Despite this, even in our institute, the utilization of PET-CT was limited to less than 80 cases over 3 years whereas we have previously reported that our center registers nearly 250 cases of GBC over a 3-year period (approximately 80 cases annually). 25

Thus, PET-CT was used in only about a third of the total cases. Selection of PET-CT as an investigative modality was driven by individual physician preferences, while the high cost of the radionuclides and longer waiting times for appointment slots were the limiting factors. In 12 (15%) cases, the investigation was recommended by a multimodality tumor board in view of equivocal findings on conventional imaging. In our study, we found out that the PET-CT scans were carried out for not only aiding in diagnosis and workup but also in evaluating response post-definitive, adjuvant or palliative therapy and in follow-up surveillance.

The ability of PET-CT to detect metastatic disease in upfront and incidental GBC ranges between 17 and 38%. 20 Also, pooled results of a recent meta-analysis indicate that PET-CT has a good sensitivity (87%) and specificity (78%) in the evaluation of primary tumor in patients with GBC. 26 27 But there is paucity of data regarding its impact on staging and follow-up of incidentally detected GBC. PET-CT was used as a staging modality in 49% of cases in our study. These patients had also undergone conventional imaging in the form of CT or MRI of the abdomen and pelvis. Twenty-six percent additional cases were upstaged as metastatic disease due to finding of FDG avid metastatic deposits apart from the primary and nodal disease. PET-CT had a substantially higher sensitivity (100%) compared to conventional imaging (41.12%) for detecting distant metastases. Thus, these patients were spared the unnecessary morbidity of radical surgery as it would not improve their overall survival. These findings also allowed more optimal utilization of surgical resources in the management of surgically salvageable patients.

**Fig. 5:**
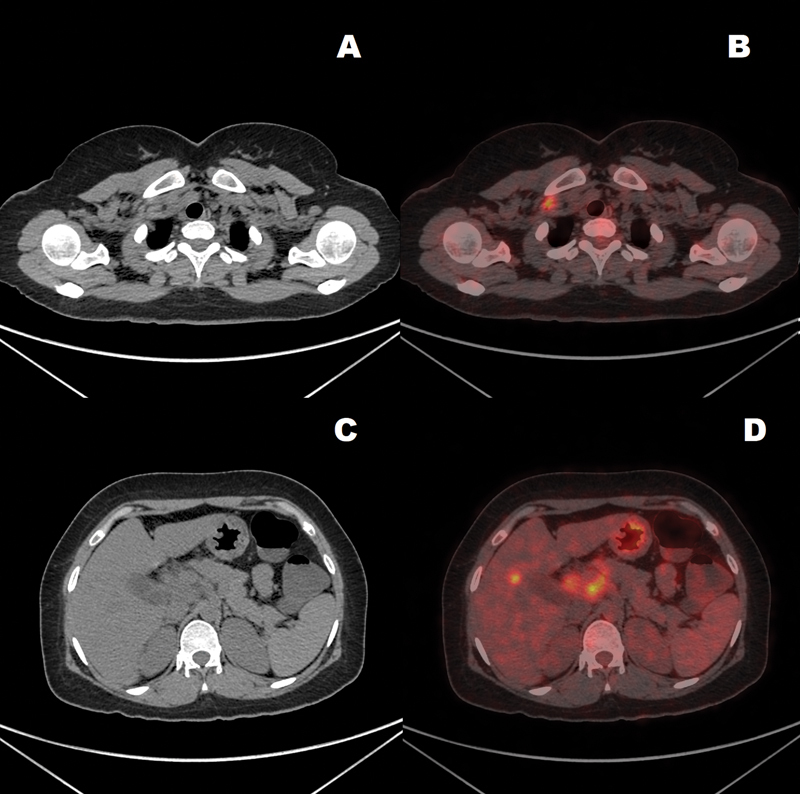
Comparison of CT with corresponding positron emission tomography-computed tomography (PET-CT) images showing additional information provided by the functional imaging. Image panel ( **A** ) shows a CT scan section with no discernible disease, while panel ( **B** ) shows PET-CT scan of the same section showing a right supraclavicular lymph node that turned out to be metastatic. Image panel ( **C** ) shows a CT scan section showing an abdominal lymph nodal mass. Panel ( **D** ) is a PET-CT scan of the same section that not only shows high metabolic activity in the lymph nodal mass, but also shows a metastatic lesion in the liver that is completely absent in the CT image.

An interesting finding was that apart from independently upstaging some patients, PET-CT was particularly useful in a specific cohort of patients who had equivocal findings on CT. In these patients, apart from the primary tumor there was only one site of suspected metastatic involvement on CECT (retroperitoneal nodes, lung nodules, or omental deposit) that was not amenable to tissue diagnoses by image-guided needle biopsy. The utilization of PET-CT confirmed high FDG avidity at these sites and also picked up other sites of metastasis that could be accessed for histopathological diagnosis thereby confirming the presence of metastatic disease and changing the final management. This was similar to a study published by Patkar et al in which around 55% of patients with equivocal findings on CT scan were upstaged by PET-CT. 28 Fig. 5 shows comparison of CT versus PET-CT in detection of liver metastases in two study cases.

PET-CT was used in 23% of patients in our study as a surveillance tool post-therapy. The scans were done for follow-up either post-surgery alone, post-NACT followed by surgery and adjuvant chemoradiotherapy, or post-surgery followed by adjuvant chemotherapy. PET-CT was able to detect 22% more patients with metastatic disease than conventional imaging in all three arms (20% vs. nil in post-surgery alone, 50% vs. nil in post-NACT plus surgery and adjuvant therapy and 53.3 vs. 33.3% in patients with surgical resection and adjuvant therapy). In detection of distant metastatic recurrences, PET-CT had a sensitivity of 100% compared to only 50% for conventional imaging. Our findings are similar to the results of studies by Chijiiwa et al 29 and Fong et al 30 in which patients with early-stage GBC (T1 and T2) who underwent radical resection showed a 5-year overall survival of 59 vs. 17% in those without radical resection. Contrary to that a higher number of patients (57%) were metastatic on follow-up in the surgical arm receiving adjuvant therapy (chemotherapy, radiation, or both). This data is in sync with several studies that show a survival rate of 5 to 15% in locally advanced GBC despite therapy. 29

Incidentally, there were no local or locoregional recurrences detected in our study population by either conventional or PET-CT imaging; hence, the ability of imaging modalities in detecting local recurrences could not be compared.

Early detection of distant recurrences in patients on surveillance ensured that they were offered palliative chemotherapy as soon as the metastatic disease was diagnosed. This was additional information gained compared to conventional imaging that changed management of these patients. However, whether the early diagnosis or treatment of metastatic disease made a difference in the overall survival of patients could not be discerned and is beyond the scope of this study.

In the group of patients who underwent PET-CT for response assessment post-chemotherapy, the two subsets taken into account in our study were post-NACT (11.1%) and post-palliative chemotherapy (88.8%). This disproportioned number ratio can be justified as GBC is known to be an aggressive malignancy with a high rate of local infiltration and metastatic spread. 31 Unfortunately due to this being a retrospective study, we did not have conventional imaging in the 18 cases considered for comparison with PET-CT. Using PET-CT alone as a tool for response assessment, we detected progressive disease in 50% of patients in the NACT subset and 56.2% in the palliative chemotherapy subset. These patients were either started on second-line chemotherapy or were offered best supportive care depending on the performance status of the patient. Only one patient in the palliative chemotherapy arm showed complete response. Patients with partial response (22.2%) and stable disease (16.6%) were continued with the line of management as before till adequate palliation was achieved.

The main limitation of our study is its retrospective and nonstatistical nature. Limited availability and cost of this modality are other limiting factors.

## Conclusion

Overall, our study showed that use of whole-body PET-CT could significantly impact the management of a large fraction of GBC patients (40%) and can be a valuable addition in the treatment of this deadly disease. However, expense and limited availability of this modality make it difficult to be incorporated in routine workup of this malignancy especially in low-middle income countries like India where it is epidemiologically dominant. It is imperative that future studies are aimed to identify the specific scenarios and roles in which this effective but expensive modality can be best utilized in this disease. Making it more accessible to patients across government oncological institutes can also help in further determining its efficacy in the above-mentioned roles.
